# Pulmonary artery reconstruction using a pulmonary vein conduit in case having an imbalanced dissection length during double-sleeve lobectomy

**DOI:** 10.1186/s44215-022-00027-w

**Published:** 2023-03-29

**Authors:** Toshiya Fujiwara, Kazuhiro Okada, Yutaka Hirano, Yuho Maki, Munehiro Saiki, Keiji Yunoki, Motoki Matsuura

**Affiliations:** 1 Department of Thoracic Surgery, Hiroshima City Hiroshima Citizens Hospital, 7-33 Motomachi, Naka-Ku, Hiroshima City, Hiroshima 730-8518 Japan; 2 Department of Cardiovascular Surgery, Hiroshima City Hiroshima Citizens Hospital, 7-33 Motomachi, Naka-Ku, Hiroshima City, Hiroshima 730-8518 Japan

**Keywords:** Lung cancer, Pulmonary artery reconstruction, Sleeve resection, Pulmonary vein conduit

## Abstract

**Background:**

Among the types of lung resection procedures, pneumonectomy carries the highest risk for mortality. In recent years, bronchovascular double-sleeve lobectomy has been performed for centrally located non-small cell lung cancer involving both the bronchus and the pulmonary artery (PA) in order to avoid pneumonectomy. The use of an autologous pulmonary vein (PV) conduit for PA reconstruction during lung-sparing resections had first been reported in 2009. Such situations may occur in cases requiring the resection of a long segment of the PA without associated bronchial sleeve resection.

**Case presentation:**

We experienced two cases who underwent PA reconstruction using a PV conduit after double-sleeve resection. In both cases, the tumor was located in the left upper lobe and invaded the long segment of the PA; however, it had not significantly invaded the bronchus. Our strategy for bronchovascular reconstruction involved the use of a PV conduit to avoid high tension on direct anastomosis given the imbalance in the excision length between the PA and the bronchus. The intraoperative and postoperative courses were uneventful, with both cases not receiving anticoagulant agents during the postoperative period. The reconstructed bronchus and PA functioned well during postoperative follow-up visits.

**Conclusions:**

Following sleeve resection, an autologous PV conduit may be indicated for PA reconstruction when an excessive distance exists between the two vascular stumps.

## Background

Among the types of lung resection procedures, pneumonectomy carries the highest risk for mortality. In recent years, bronchovascular double-sleeve lobectomy has been performed for centrally located non-small cell lung cancer (NSCLC) involving both the bronchus and the pulmonary artery (PA) in order to avoid pneumonectomy. The use of an autologous pulmonary vein (PV) conduit for the reconstruction of the PA in lung-sparing resections had first been reported in 2009 by Cerezo et al. [1]. Such situations may occur in cases requiring the resection of a long PA segment without associated bronchial sleeve resection. Our study is the first report of PA reconstruction using a PV conduit after extended double-sleeve resection due to imbalance in the excision length between the PA and bronchus.

## Case presentation

### Case 1

**Fig. 1:**
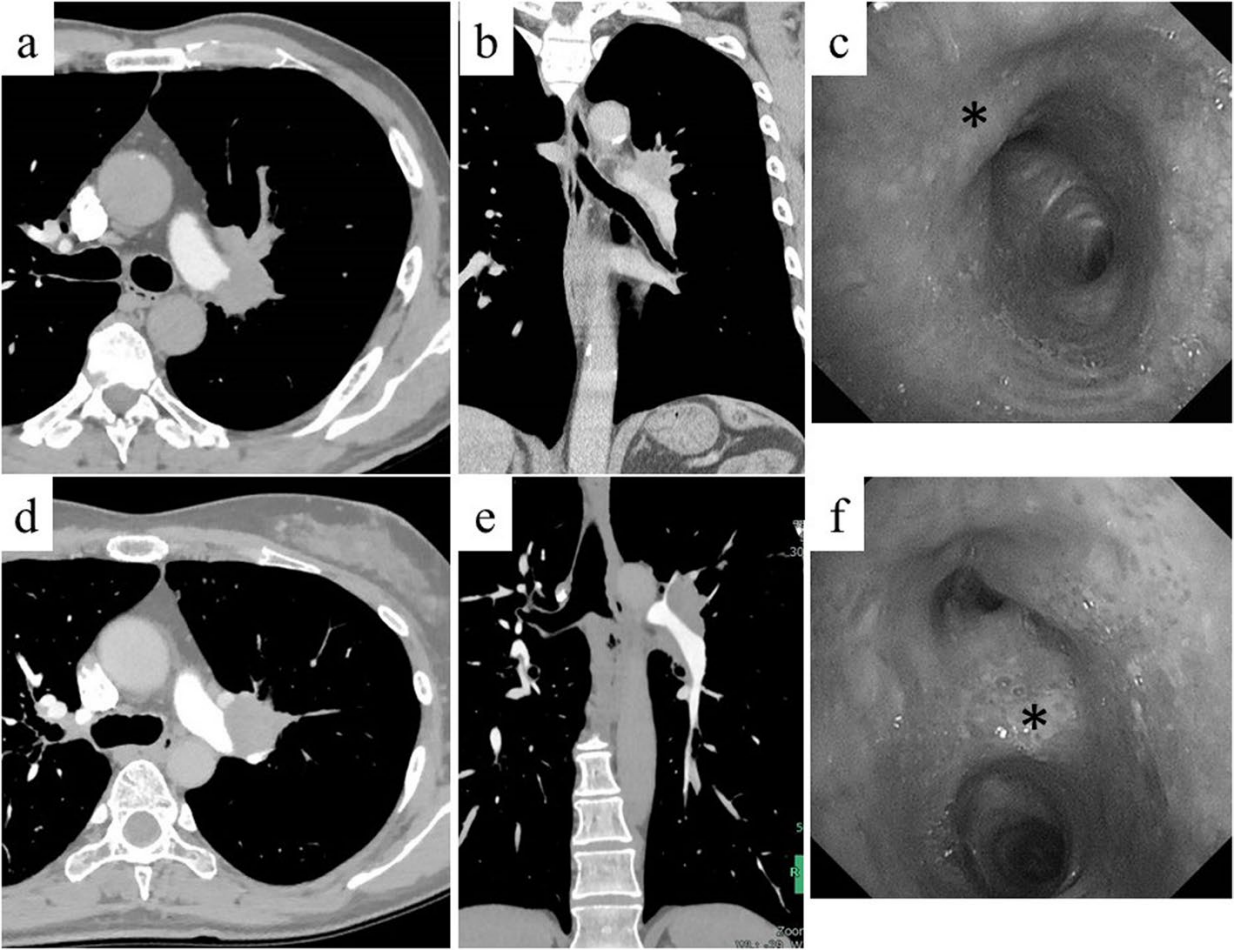
Chest computed tomography showing the pulmonary hilar tumor in the left upper lobe. The tumor was suspected to have invaded the long segment of the PA (**a**, **b** case 1; **d**, **e** case 2). Bronchoscopy showed the exposition of the tumor to the left upper bronchus. The tumor was diagnosed as squamous cell carcinoma by TBB from * site (**c** case 1—orifice of left upper bronchus; **f** case 2—second carina)

A 65-year-old man with mild dyspnea was referred to our department. He had a history of type 2 diabetes mellitus, distal pancreatectomy for pancreatic cancer followed by adjuvant chemotherapy 6 years prior to presentation, and smoking 35 pack-years until 10 years prior to presentation. He presented with a pulmonary hilar tumor with a diameter of approximately 42 mm in the left upper lobe on chest computed tomography (CT) (Fig. 1a, b). The tumor was suspected to have invaded the long segment of the PA. Positron emission tomography (PET) showed high accumulation of fluorodeoxyglucose (FDG) in the tumor; however, no accumulation in regional lymph nodes and distant organs was noted. On bronchoscopy, exposition of the tumor was observed in the left upper bronchus, which was subsequently diagnosed as squamous cell carcinoma following transbronchial biopsy (TBB) (Fig. 1c). We considered surgical resection of the predicted cT3 N1 M0 stage IIIA tumor.

### Case 2

A 49-year-old woman complaining of persistent cough and bloody sputum visited our hospital. She had a history of extirpation for a pleomorphic adenoma of the salivary gland 7 years prior to presentation and is a current smoker with 29 pack-year. Chest CT revealed a pulmonary hilar tumor with a diameter of approximately 55 mm in the left upper lobe with obstructive pneumonia (Fig. 1d, e). The tumor was suspected to have invaded the long segment of the PA. Increased accumulation of FDG was observed only in the tumor via PET. Following TBB, the tumor was diagnosed as squamous cell carcinoma (Fig. 1f). Surgical resection of the predicted cT3 N0 M0 stage IIB tumor was then planned.

### Clinical course and operative procedure

**Fig. 2:**
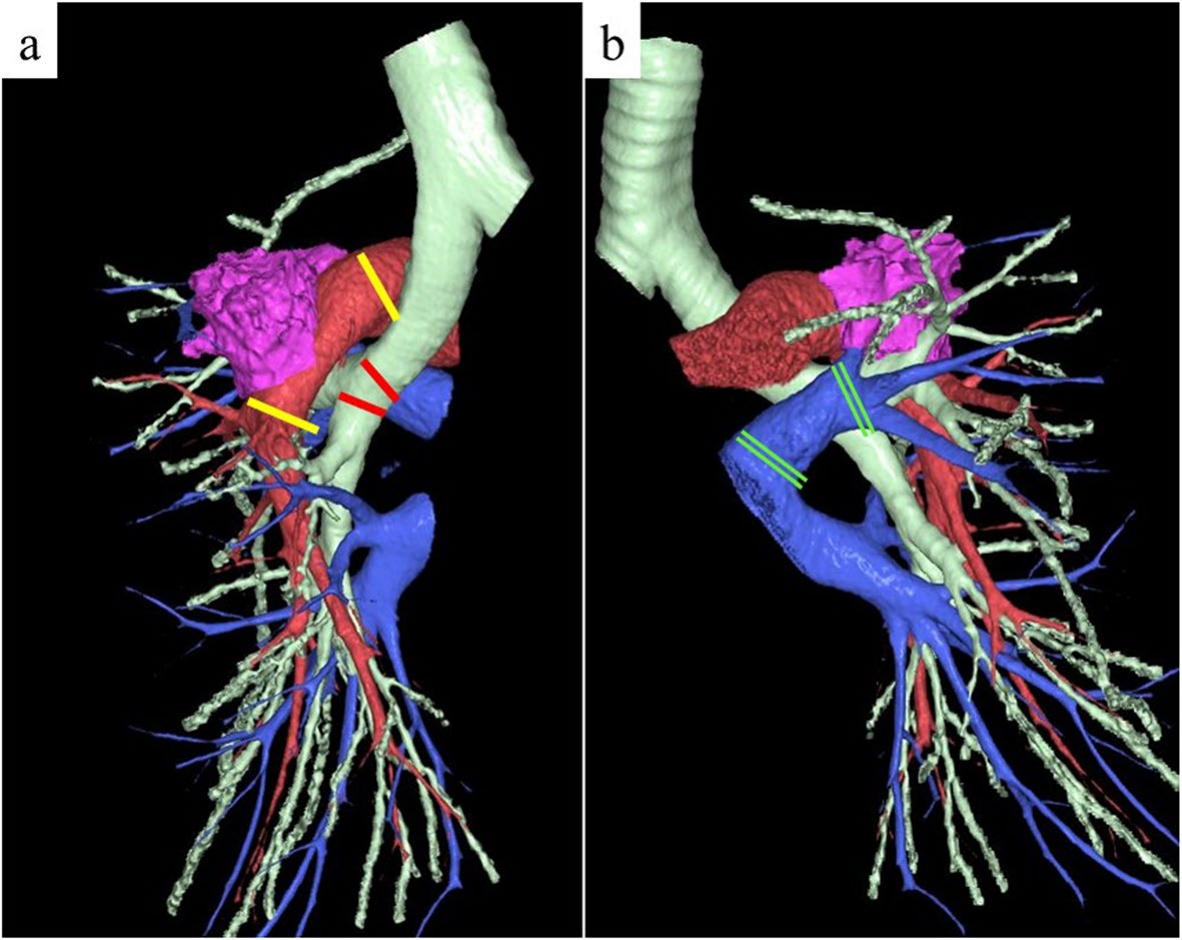
Preoperative three-dimensional computed tomography angiography of case 1. We anticipated that the length of the PA dissection would be longer than that of the bronchial dissection **a** and a 25–30 mm section of the left superior PV could be retrieved for an autologous conduit **b**

After discussing the surgical strategy for both cases, we planned to preserve the lung parenchyma using the double-sleeve resection technique and reconstruction; however, we anticipated that the length of the PA dissection would be longer than that of the bronchial dissection (Fig. 2a). Based on CT measurement, the predicted dissection length of the PA was 3.9 cm for case 1 and 3.8 cm for case 2. Thus, our strategy for bronchovascular reconstruction involved the use of a PV conduit to avoid high tension on direct anastomosis and resolve the imbalance in excision length between the PA and the bronchus. Preoperative CT confirmed that a 25–30-mm section of the left superior PV could be retrieved for an autologous graft in both cases (Fig. 2b).

In case 1, the tumor in the hilum of the left upper lobe showed extensive invasion from the central side of A3 to just above the orifice of A6 and directly involved the left vagal nerve and subaortic lymph node, while the bronchus slightly infiltrated the inlet of the left upper bronchus. In case 2, the tumor in the hilum of the left upper lobe involved A3 and extended to just above the orifice of A6, while the tumor invasion to the bronchus was over the second carina but not to the central side. In both cases, all margins were examined on frozen sections and confirmed to be negative for cancer invasion before anastomosis.

**Fig. 3:**
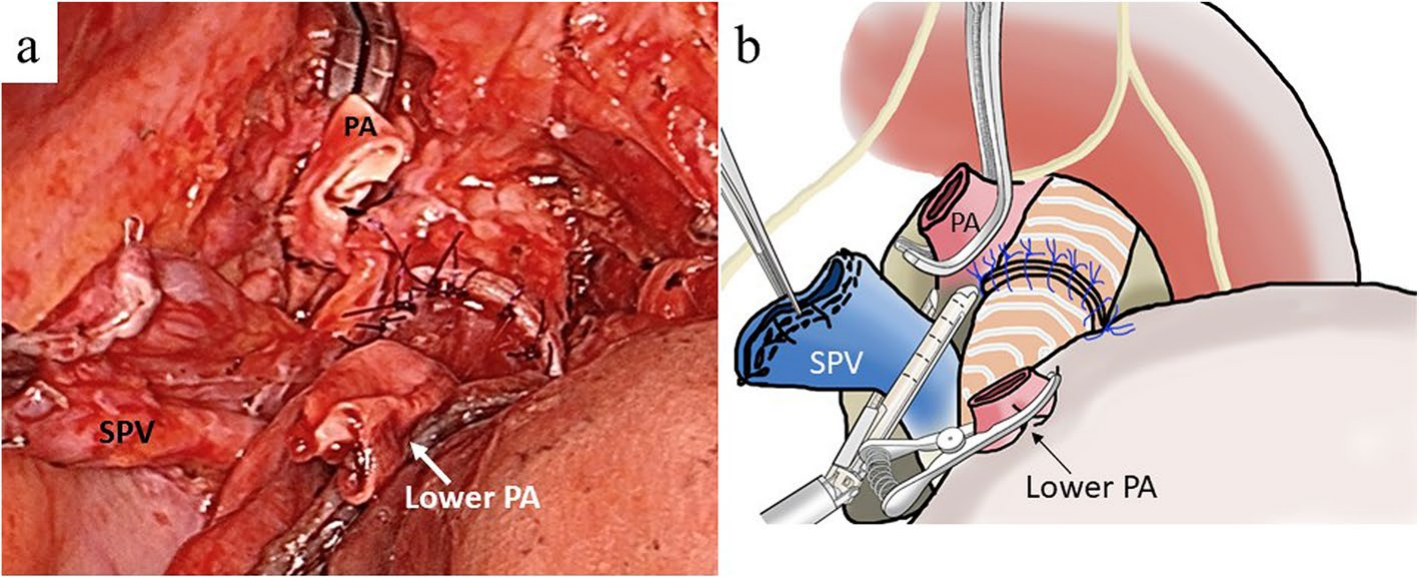
Operative view **a** and schema **b** of case 2 after anastomosis of bronchus. The superior pulmonary vein could be retrieved using a length of ≥ 25 mm. SPV, superior pulmonary vein

**Fig. 4:**
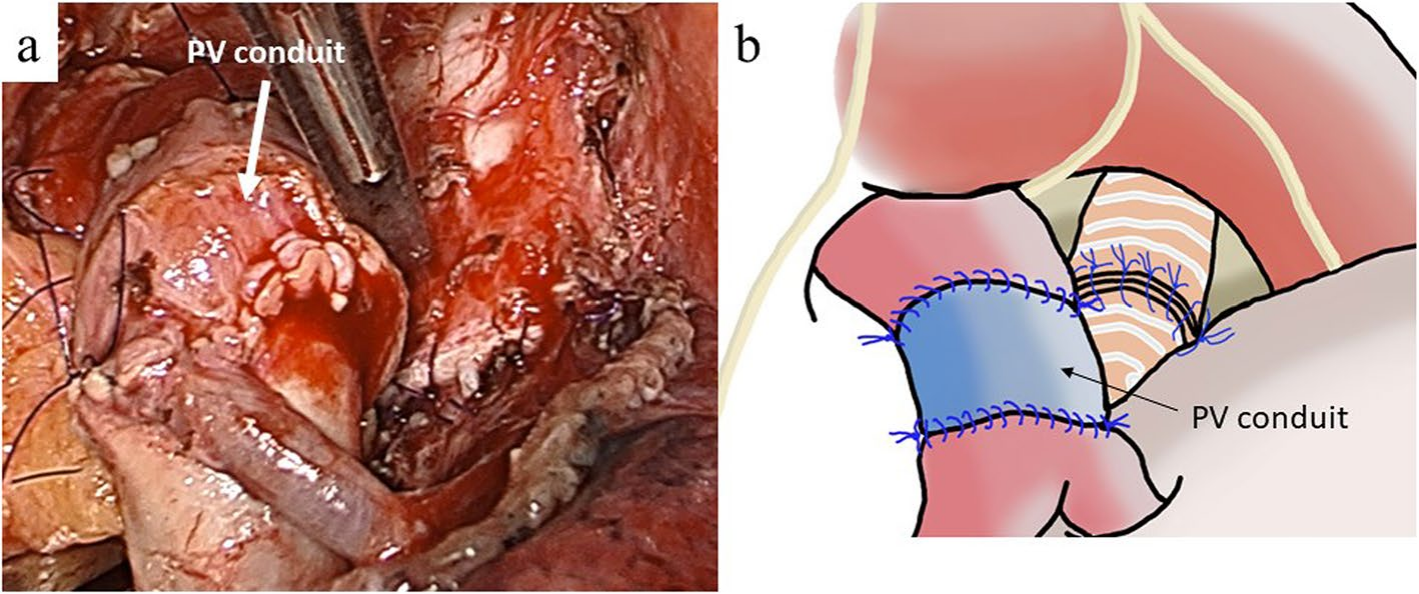
Operative view **a** and schema **b** of case 1 after PA reconstruction using the PV conduit

**Fig. 5:**
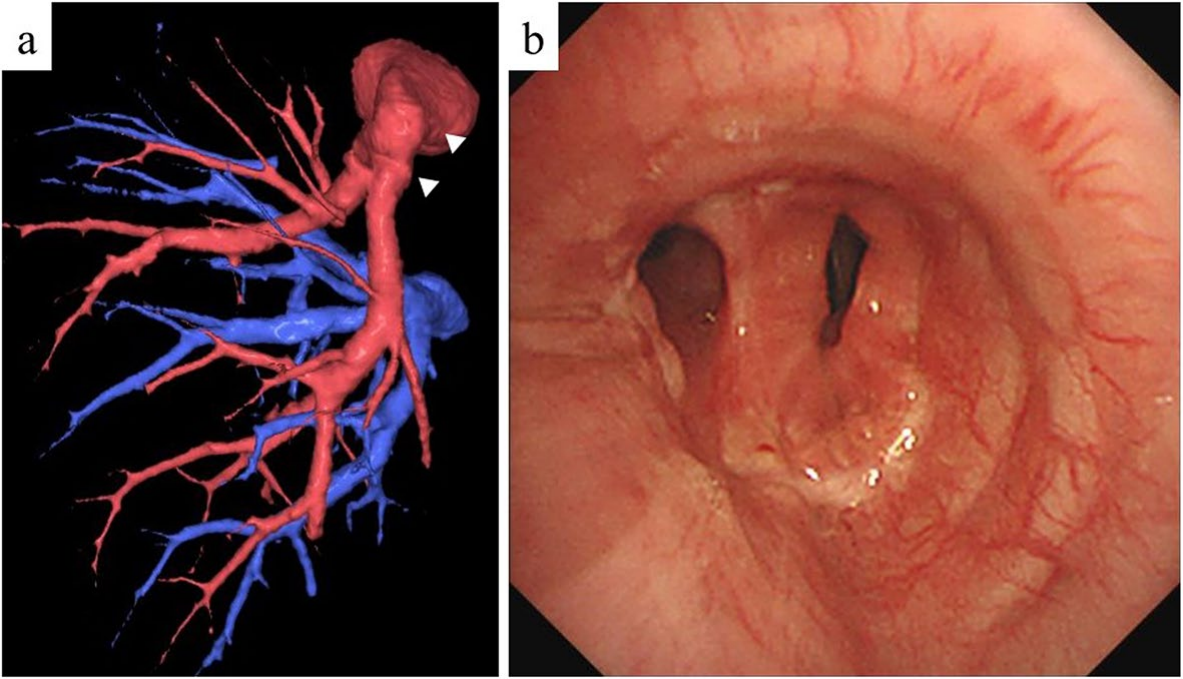
Postoperative findings of case 1. **a** Three-dimensional computed tomography angiography on postoperative day 4. Arrowheads indicate anastomosis of the PA and PV conduit. **b** Bronchoscopy on postoperative day 32 showed an anastomosis between the left main bronchus and the lower bronchus

We performed double-sleeve lobectomy along with systematic lymph node dissection via left posterolateral thoracotomy through the 4th intercostal space. The PA segment to be resected needed to be longer than the bronchus. An incision was made in the pericardium below the superior PV to open the pericardial cavity. We then confirmed that ≥ 25 mm of the superior pulmonary vein could be retrieved (Fig. 3a, b). If the superior pulmonary vein of sufficient length is not available, the pericardium is used to fill the defect. The pericardium can be easily harvested of any size. Pneumonectomy is also an option, but I prefer to avoid it as much as possible. Bronchial reconstruction was performed using the hybrid anastomotic technique, which involved running sutures in the posterior wall and interrupted sutures in the anterior wall using 4–0 absorbable monofilament sutures. Running 5–0 non-absorbable monofilament sutures were used for vascular reconstruction, starting from the central side (Fig. 4a, b). Since the first anastomosis can be observed all around, it is easy to confirm the anastomosis on the dorsal side. The second anastomosis is extended by the PV conduit, and the anastomosis is relatively flexible. During the reconstruction, heparin sodium was administered systemically, and the activated whole blood clotting time was controlled to around 250 s. After reconstruction, each anastomosis was isolated using a pedunculated thymic flap. The feature of this surgical procedure is to keep the bronchial anastomoses as stress-free as possible. The pulmonary ligament was cut by clamping the inferior pulmonary vein, but no pericardiotomy was required. Surgery time and PA clamping time were 521 min and 139 min in case 1 and 394 min and 122 min in case 2. The amount of blood loss was 340 g in case 1 and 260 g in case 2. No anticoagulant agents were administered throughout the postoperative period. In both cases, the intraoperative and postoperative courses were uneventful. The histopathological staging was pT4 N2 M0 stage IIIB in case 1 and pT3 N1 M0 stage IIIA in case 2, and the tumor was extensively invaded into PA in both cases. The reconstructed bronchus and PA functioned well during postoperative follow-up visits (Fig. 5a, b). The patients received adjuvant chemotherapy and were doing well 58 (case 1) and 50 (case 2) months after surgery without recurrence.

## Discussion and conclusions

Pneumonectomy has been considered a disease in itself given that it causes cardiac stress, can be fatal among patients who have pneumonia in the dependent lung, and decreases quality of life. Surgeons have strived to preserve the lung parenchyma as much as possible while securing oncological curability. In recent years, several reports have been published on centrally located lung cancer in which the lungs were preserved via bronchovascular reconstruction [2–5]. Pneumonectomy is unavoidable in patients who have extensive cancer invasion into the bronchus; however, should the cancer infiltration primarily involve the PA, various methods are available for reconstructing it.

The reconstruction method depends on the extent of tumor infiltration into the PA. When only minor PA defect is present, direct closure or patch compensation can be sufficient; however, extensive defects would warrant circumferential resection, which complicates the reconstruction method. In more locally advanced NSCLC patients, reports have suggested the aggressive application of bronchovascular plastic procedures to avoid pneumonectomy. Extended sleeve lobectomy was initially described by Johnston and colleagues in 1959, as well as by Okada et al. in 1999 [6, 7]. This technique is an alternative procedure to remove more than one lobe using the bronchovascular plastic technique in patients with locally advanced lung cancer. Okada et al. classified extended sleeve lobectomy into three groups, types A, B, and C, according to the mode of reconstruction [7]. Later, Berthet et al. added type D extended sleeve lobectomy [8]. The aforementioned techniques are feasible and have shown good local control and long-term survival. Angioplasty is often encountered in types A and B [7]. These procedures assume that the lengths of bronchial and PA dissection are equivalent.

In many cases of extended double-sleeve resection, end-to-end anastomosis is often possible given the moderate distance between each stump. Imbalances in the excision length between the PA and the bronchus, as in our cases, warrant countermeasures to avoid pneumonectomy, such as additional bronchial resection or interposition connecting the stumps of the PA. Given that the former may increase the risk of anastomotic failure and may require transposition of the PV with a risk of fatal complications, care should be exercised when selecting this approach. The latter requires some ingenuity in the procedure, although the method remains significant.

There have been some reports regarding conduit interposition of the PA. When performing conduit interposition, prosthetic materials are required. There are two types of prosthetic materials: a synthetic material and a biological material. Synthetic materials, which are mainly made from polyethylene terephthalate (Dacron) and polytetrafluoroethylene (PTPE) [9], carry a high risk for thrombosis and infection and need long-term anticoagulation. Biological prostheses, which can include the pericardium, azygos vein, great saphenous vein, superficial femoral vein, cryopreserved allograft, bovine pericardium, and PV, have some advantages over synthetics in terms of biocompatibility while having lesser risk of infections and thrombosis. These prostheses have different characteristics depending on the material. Among them, the autologous pericardium is the most frequently used [10–12]. An autologous pericardium has adequate thickness and resistance, is free of cost, and is easily available on both sides of the thoracic cavity. However, some disadvantages may include shrinking and curling, as well as technical difficulties in adaptation and suturing. These technical limits can be resolved by fixing the pericardial grafts using glutaraldehyde-buffered solution at the cost of potential tissue damage and calcification [13, 14]. Bovine pericardium allows for tailor-made conduits and is useful when the PA defect is longer [15]; however, preparing these conduits is time-consuming. The great saphenous vein and superficial femoral vein require an additional operative field with repositioning [16]. Collection of azygos vein is limited to right-sided procedures [17]. In addition, we believe that these materials do not fit the caliber of the pulmonary artery trunk and are not rigid enough. Although cryopreserved allogeneic grafts are covered by insurance for some limited treatments in Japan, they are not common and are not easily available [18, 19].

Shimizu et al. developed a selection criteria chart for PA reconstruction and conduit material. If the PA defect is less than 2.5 without hilar invasion, the PV conduit is an option. If the PA defect is more than 2.5 cm, it indicates conduit reconstruction with bovine pericardium or synthetic material. End-to-end anastomosis is indicated for double-sleeve resection. However, although our cases had defect of length more than 2.5 cm, they are rare cases having imbalanced dissection length between the PA and bronchus and are considered to be excluded from this criterion. Since the bronchial dissection length is about 1 cm, we determined that the PV conduit was just right to compensate for the defect length.

The use of an autologous PV conduit for the reconstruction of the PA in lung-sparing resections had first been reported in 2009 by Cerezo and colleagues [1]. Cerezo et al. reported the resection and reconstruction of the PA without bronchial resection [1]. In line with this, we applied their technique to double-sleeve resection. The PV is originally a tubular structure that can be used without molding and has the optimal caliber, length, and stiffness. However, there are some limitations with regard to its use. To harvest the PV conduit, the pericardial cavity needs to be opened, which is the last line of defense during reoperation. Only the left superior pulmonary vein is sufficient for the length of the PV conduit; however, while sleeve resection is often required for left lung cancer, they are rarely used for the right-sided cancer. The PV cannot be used as a conduit when cancer infiltration is present. If necessary, frozen sections of PV margins should be used to confirm negativity for cancer invasion. Our cases had tumors on the dorsal side with an intact PV. Since it is an autologous tissue, heparin sodium is used only during surgeries and is unnecessary for postoperative anticoagulant therapy. Cerezo et al. found that PV conduit-specific complications, including thrombosis and leakage, can be expected in < 5% of cases [1]. Puma et al. and D’Andrilli et al. reported no cases of local recurrence at the PV graft site, indicating that the technique is oncologically reliable [20–22].

In recent years, there have been reports on autologous lung transplantation for lung cancer using lung transplantation techniques [23, 24]. This procedure has several advantages such as the resection on the back-table, favorable field of view, no bleeding, safe surgical margins, and appropriate lung preservation to avoid warm ischemic damage [23]. However, lung transplantation is a procedure that should be limited to organ transplantation facilities given the difficulty of performing it as a general medical treatment. Moreover, there is also a concern regarding the cost of preservative solution.

In conclusion, reconstruction of the PA with autologous PV conduit will prove to be an excellent approach when an excessive distance exists between the two vascular stumps.

## Data Availability

Not applicable.

## Abbreviations

PA: Pulmonary artery
PV: Pulmonary vein
NSCLC: Non-small cell lung cancer
CT: Computed tomography
PET: Positron emission tomography
FDG: Fluorodeoxyglucose
TBB: Transbronchial biopsy
PTPE: Polytetrafluoroethylene

## References

[1] Cerezo F, Cano JR, Espinosa D, Salvatierra A. New technique for pulmonary artery reconstruction. Eur J Cardiothorac Surg. 2009;36:422–3. 19501519 10.1016/j.ejcts.2009.03.060

[2] Rendina EA, Venuta F, Ciriaco P, Ricci C. Bronchovascular sleeve resection. Technique, perioperative management, prevention, and treatment of complications. J Thorac Cardiovasc Surg. 1993;106:73–9. 8321007

[3] Rendina EA, De Giacomo T, Venuta F, Ciccone AM, Coloni GF. Lung conservation techniques: bronchial sleeve resection and reconstruction of the pulmonary artery. Semin Surg Oncol. 2000;18:165–72. 10657918 10.1002/(sici)1098-2388(200003)18:2<165::aid-ssu10>3.0.co;2-m

[4] Ma Z, Dong A, Fan J, Cheng H. Does sleeve lobectomy concomitant with or without pulmonary artery reconstruction (double sleeve) have favorable results for non-small cell lung cancer compared with pneumonectomy? A meta-analysis. Eur J Cardiothorac Surg. 2007;32:20–8. 17442581 10.1016/j.ejcts.2007.03.018

[5] D’Andrilli A, Venuta F, Maurizi G, Rendina EA. Bronchial and arterial sleeve resection after induction therapy for lung cancer. Thorac Surg Clin. 2014;24:411–21. 25441134 10.1016/j.thorsurg.2014.07.006

[6] Johnston JB, Jones PH. The treatment of bronchial carcinoma by lobectomy and sleeve resection of the main bronchus. Thorax. 1959;14:48–54. 13635645 10.1136/thx.14.1.48 PMC1018472

[7] Okada M, Tsubota N, Yoshimura M, Miyamoto Y, Matsuoka H, Satake S, et al. Extended sleeve lobectomy for lung cancer: the avoidance of pneumonectomy. J Thorac Cardiovasc Surg. 1999;118:710–4. 10504638 10.1016/S0022-5223(99)70017-6

[8] Berthet J-P, Paradela M, Jimenez MJ, Molins L, Gómez-Caro A. Extended sleeve lobectomy: one more step toward avoiding pneumonectomy in centrally located lung cancer. Ann Thorac Surg. 2013;96:1988–97. 24035301 10.1016/j.athoracsur.2013.07.011

[9] Yoshida K, Toishi M, Agatsuma H, Kumeda H, Eguchi T, Terada Y, Shiina T. Pulmonary artery reconstruction with a prosthetic conduit in lung cancer. Ann Thorac Cardiovasc Surg. 2014;20(Suppl):505–8. 24835921 10.5761/atcs.cr.14-00009

[10] Rendina EA, Venuta F, De Giacomo T, Vizza DC, Ricci C. Reconstruction of the pulmonary artery by a conduit of autologous pericardium. J Thorac Cardiovasc Surg. 1995;110:867–8. 7564463 10.1016/S0022-5223(95)70128-1

[11] Rendina EA, Venuta F, De Giacomo T, Ciccone AM, Moretti M, Ruvolo G, et al. Sleeve resection and prosthetic reconstruction of the pulmonary artery for lung cancer. Ann Thorac Surg. 1999;68:995–1001. 10509997 10.1016/s0003-4975(99)00738-9

[12] Venuta F, Ciccone AM. Reconstruction of the pulmonary artery. Semin Thorac Cardiovasc Surg. 2006;18:104–8. 17157228 10.1053/j.semtcvs.2006.05.004

[13] D’Andrilli A, Ibrahim M, Venuta F, De Giacomo T, Coloni GF, Rendina EA. Glutaraldehyde preserved autologous pericardium for patch reconstruction of the pulmonary artery and superior vena cava. Ann Thorac Surg. 2005;80:357–8. 15975412 10.1016/j.athoracsur.2004.02.012

[14] Isenburg JC, Simionescu DT, Vyavahare NR. Tannic acid treatment enhances biostability and reduces calcification of glutaraldehyde fixed aortic wall. Biomaterials. 2005;26:1237–45. 15475053 10.1016/j.biomaterials.2004.04.034

[15] Shimizu K, Nagashima T, Ohtaki Y, Takahashi T, Mogi A, Kuwano H. Pulmonary artery reconstruction with a tailor-made bovine pericardial conduit following sleeve resection of a long segmental pulmonary artery for the treatment of lung cancer: technical details of the dog-ear method for adjusting diameter during vascular anastomosis. Gen Thorac Cardiovasc Surg. 2017;65:304–7. 27796918 10.1007/s11748-016-0725-1

[16] Yoshimi F, Amemiya R, Asato Y, Koizumi S, Hasegawa H, Matsueda K, et al. Pulmonary artery reconstruction using a great saphenous vein autograft in the treatment of bronchogenic cancer. Surg Today. 1994;24:570–3. 7919746 10.1007/BF01884583

[17] Xuegang L, Chao S, Zhen T, Xiaojun L, Ge L, Lei Z. Pulmonary artery reconstruction using autologous pericardium or azygos venae substitute for surgical treatment of central non-small cell lung cancer. Cell Biochem Biophys. 2013;67:949–55. 23549737 10.1007/s12013-013-9588-6

[18] Berthet JP, Boada M, Paradela M, Molins L, Matecki S, Marty-Ané CH, et al. Pulmonary sleeve resection in locally advanced lung cancer using cryopreserved allograft for pulmonary artery replacement. J Thorac Cardiovasc Surg. 2013;146:1191–7. 23953718 10.1016/j.jtcvs.2013.07.003

[19] Kitamura S, Yagihara T, Kobayashi J, Nakajima H, Toda K, Fujita T, et al. Mid- to long-term outcomes of cardiovascular tissue replacements utilizing homografts harvested and stored at Japanese institutional tissue bank. Surg Today. 2011;41:500–9. 21431482 10.1007/s00595-010-4459-x

[20] Puma F, Capozzi R, Daddi N, Ragusa M, Cagini L, Quintili A, et al. Experience with the autologous pulmonary vein for pulmonary arterioplasty. Eur J Cardiothorac Surg. 2011;40:e107–11. 21680195 10.1016/j.ejcts.2011.05.012

[21] D’Andrilli A, Maurizi G, Andreetti C, Ciccone AM, Ibrahim M, Poggi C. Pulmonary artery reconstruction with pulmonary vein conduit for lung cancer: medium-term results. Ann Thorac Surg. 2014;98:990–5. 25038016 10.1016/j.athoracsur.2014.04.110

[22] D’Andrilli A, Maurizi G, Cicconea AM, Andreettia C, Ibrahima M, Menna C. Long-segment pulmonary artery resection to avoid pneumonectomy: long-term results after prosthetic replacement. Eur J Cardiothorac Surg. 2018;53:331–5. 29029026 10.1093/ejcts/ezx353

[23] Tanaka S, Sugimoto S, Soh J, Oto T. Long-term outcomes of pneumonectomy, back-table lung preservation, double-sleeve resection and reimplantation for advanced central lung cancer: the Oto procedure. Eur J Cardiothorac Surg. 2019;56:213–4. 10.1093/ejcts/ezy431 30590487

[24] Nakajima D, Ohsumi A, Hamaji M, Chen-Yoshikawa TF, Date H. Expanded indications for auto-lung transplant technique. Gen Thorac Cardiovasc Surg. 2020;68:828–32. 31939102 10.1007/s11748-020-01289-3

